# Vestibular dysfunction in acute traumatic brain injury

**DOI:** 10.1007/s00415-019-09403-z

**Published:** 2019-06-14

**Authors:** Hani J. Marcus, Heidi Paine, Matthew Sargeant, Susie Wolstenholme, Katie Collins, Natalie Marroney, Qadeer Arshad, Kevin Tsang, Brynmor Jones, Rebecca Smith, Mark H. Wilson, Heiko M. Rust, Barry M. Seemungal

**Affiliations:** 1 Imperial College Healthcare NHS Trust, London, UK; 2 Brain and Vestibular Group (BAVG), Neuro-Otology Unit, Imperial College London, Room 10L 17, Charing Cross Campus, London, W6 8RF UK

**Keywords:** Head trauma, Head injury, Concussion, Dizziness, Vertigo

## Abstract

Traumatic brain injury (TBI) is the commonest cause of disability in under-40-year-olds. Vestibular features of dizziness (illusory self-motion) or imbalance which affects 50% of TBI patients at 5 years, increases unemployment threefold in TBI survivors. Unfortunately, vestibular diagnoses are cryptogenic in 25% of chronic TBI cases, impeding therapy. We hypothesized that chronic adaptive brain mechanisms uncouple vestibular symptoms from signs. This predicts a masking of vestibular diagnoses chronically but not acutely. Hence, defining the spectrum of vestibular diagnoses in acute TBI should clarify vestibular diagnoses in chronic TBI. There are, however, no relevant acute TBI data. Of 111 Major Trauma Ward adult admissions screened (median 38-years-old), 96 patients (87%) had subjective dizziness (illusory self-motion) and/or objective imbalance were referred to the senior author (BMS). Symptoms included: feeling unbalanced (58%), headache (50%) and dizziness (40%). In the 47 cases assessed by BMS, gait ataxia was the commonest sign (62%) with half of these cases denying imbalance when asked. Diagnoses included BPPV (38%), acute peripheral unilateral vestibular loss (19%), and migraine phenotype headache (34%), another potential source of vestibular symptoms. In acute TBI, vestibular signs are common, with gait ataxia being the most frequent one. However, patients underreport symptoms. The uncoupling of symptoms from signs likely arises from TBI affecting perceptual mechanisms. Hence, the cryptogenic nature of vestibular symptoms in TBI (acute or chronic) relates to a complex interaction between injury (to peripheral and central vestibular structures and perceptual mechanisms) and brain-adaptation, emphasizing the need for acute prospective, mechanistic studies.

## Introduction

Traumatic brain injury (TBI) is the commonest cause of disability in the under 40-year-olds and persisting imbalance and dizziness is an independent predictor of unemployment at 6 months [1]. Since half of TBI patients at 5 years have vestibular complaints [2], the socioeconomic impact is considerable. Providing effective treatment requires accurate diagnosis, however, a quarter of chronic dizziness post-TBI is cryptogenic [3].

Brain adaption, both beneficial and disadvantageous to recovery, may mask the features of vestibular dysfunction in chronic patients. Hence, studying vestibular dysfunction in acute TBI—where chronic adaptive mechanisms have not developed—may clarify the mechanisms of chronic post-TBI dizziness. We hypothesize that acute TBI impairs balance by simultaneously affecting peripheral (inner ear) and central (brain) components of the balance system. This is supported by similar findings in chronic TBI patients [4]. The finding that post-TBI dizziness relates to specific vestibular diagnoses would support the rationale for controlled studies assessing whether active assessment and treatment of vestibular diagnoses in acute TBI accelerate recovery.

## Methods

### Study design

Patients with acute TBI admitted to our local Major Trauma Ward (MTW) were screened by therapists (SW, KC, and NM) and patients with vestibular symptoms and/or signs were referred to BMS (who provides an acute neurological service to the trauma ward). The data represent an audit of TBI patients screened by the therapists in office hours, hence, not all trauma ward cases were included (e.g., those with a brief weekend admission). All patients had brain imaging (usually CT scanning) and these scans were reviewed by a neuro-radiologist.

### Study setting and participants

All adult Major Trauma Ward admissions screened by the therapists between June 2014 and May 2015 were included. A neuro-otological referral was made if patients reported vestibular symptoms assessed via a 2-minute screening questionnaire. Patients were also referred if they displayed signs of postural/gait instability irrespective of their symptoms. Patient data were obtained as standard of care for patients referred for a neuro-otology opinion. The review of these data was as approved by the local institutional research ethics process.

### Neuro-otological evaluation

Clinical assessment included ophthalmoscopy and otoscopy, eye movements (cover test, gaze testing, saccades, smooth pursuit, vestibular ocular reflex testing via the head impulse test), Hallpike manoeuvre and gait assessment [Romberg test (20 s), tandem walking and tandem stance]. Where possible, clinical signs were recorded via laboratory testing (e.g., video or electro-oculography, rotational chair and otolith testing).

### Outcomes and statistical analysis

The following were recorded: (1) demographics, (2) presenting symptoms, (3) examination findings, and (4) clinical diagnoses. Data were analyzed using SPSS 22.0 (IBM, New York, USA). The frequencies of patients’ symptoms, signs, and final diagnoses, were reported.

## Results

Screening 111 patients showed 96 (87%) with vestibular symptoms (e.g., feeling unbalanced or illusory self- or environmental motion) or vestibular signs of gait or postural ataxia or ocular motor signs of peripheral or central vestibular dysfunction (e.g., spontaneous or positional vestibular nystagmus, positive head impulse).

About half of cases were discharged prior to evaluation by BMS who personally assessed 47 of the 96 patients identified by the therapists’ screening, as having vestibular dysfunction.

The median age of the 96 patients was 38 years (range: 17–100) with a male: female ratio of 2.7:1. Patients presented with moderate-to-severe [Mayo criteria]; [5] TBI following road traffic accidents (30/96; 31%), falls (30/96; 31%) or assault (13/96; 14%), with a median admission GCS of 15 (range 3–15).

In patients assessed by BMS, the commonest signs and diagnoses are depicted in Table 1. Although the presence or absence of a gait ataxia was always recorded, we did not distinguish between a cerebellar gait ataxia or a vestibular gait ataxia. A vestibular gait ataxia, was first described by Brandt et al. [6] in patients with an acute peripheral vestibular loss—e.g., in vestibular neuritis—where patients display better balance walking (including tandem walking) and worse balance standing still. In contrast, patients with a cerebellar ataxia are worse walking (especially tandem walking, quantified by assessing errors out of 10 tandem steps [7]) and better standing still. We also observed vestibular ataxia in patients with preserved peripheral vestibular function (assessed by the clinical head impulse test and fundoscopic gaze assessment). Notably, half of the patients who showed clear gait ataxia denied feelings of imbalance when walking (14/29; 48%) (Table 1).

**Table 1:** Clinical findings in numbers and percentage in 47 TBI patients

Clinical findings	Count	% of *N* (%)
Gait ataxia	29	62
Gait ataxia without complaints	14	30
Benign paroxysmal positional vertigo (BPPV)	18	38
Headache + photo/– phonophobia	16	34
Acute unilateral peripheral vestibular loss	9	19
Temporal bone fracture	6	13

**Table 2:**
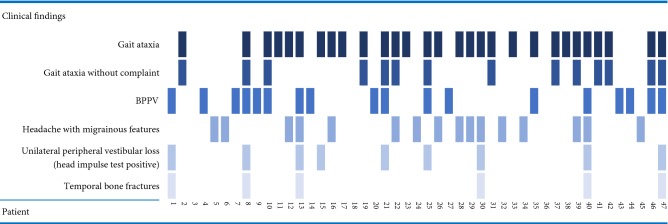
Clinical signs, findings and symptoms in 47 TBI patients

## Discussion

### Principal findings

Vestibular dysfunction is common in acute TBI patients typically combining peripheral and central vestibular diagnoses (Table 2)—as we found in chronic TBI patients with persisting vestibular symptoms [4]. That TBI patients have additional neurological conditions (e.g., cognitive dysfunction and epilepsy) indicates a complex neurological need that is poorly served as neurologists are currently not routinely involved in acute TBI care [8].

Half of the cases with clinically obvious gait ataxia reported no balance problem which may lead to an under-recognition of vestibular dysfunction by clinical staff in acute TBI. This replicates a recent finding in a smaller group of acute TBI patients (with intracranial abnormalities on CT), where, using a sports concussion tool, we found no correlation between objective signs and symptoms of imbalance [9].

Although common, we cannot yet advocate the routine screening and treatment of vestibular conditions in acute TBI since there is no evidence base for such intervention. For example, a retrospective study [10] found a recurrence rate of 67% with traumatic BPPV versus only 14% with idiopathic BPPV, which could support treating BPPV in the subacute phase (when it may respond to treatment) and not the acute phase (when it may recur).

An acute unilateral peripheral vestibular loss was found in 20% of cases, two thirds with a petrous temporal bone fracture and one third of uncertain aetiology. Theoretically, the simultaneous injury of peripheral and central vestibular structures, relatively unique to acute TBI, could worsen clinical recovery.

### Limitations

The single-unit setting limits the generalisability of our findings to other units, although our finding of multiple vestibular diagnoses in individual patients supports a similar finding in an outpatient case series [4].

## Conclusions

Central and peripheral vestibular dysfunction—often in combination—is common in acute TBI. Prospective studies are required to assess whether acute intervention improves patient outcomes.
